# Supplementary material for: CASB: a concanavalin A‐based sample barcoding strategy for single‐cell sequencing
Source: Mol Syst Biol. 2021 Apr 6;17(4):e10060. doi: 10.15252/msb.202010060 (PMC8022202; doi:10.15252/msb.202010060)
Supplement: Supplementary file 1 — Expanded View Figures PDF [file MSB-17-e10060-s002.pdf]

Expanded View Figures

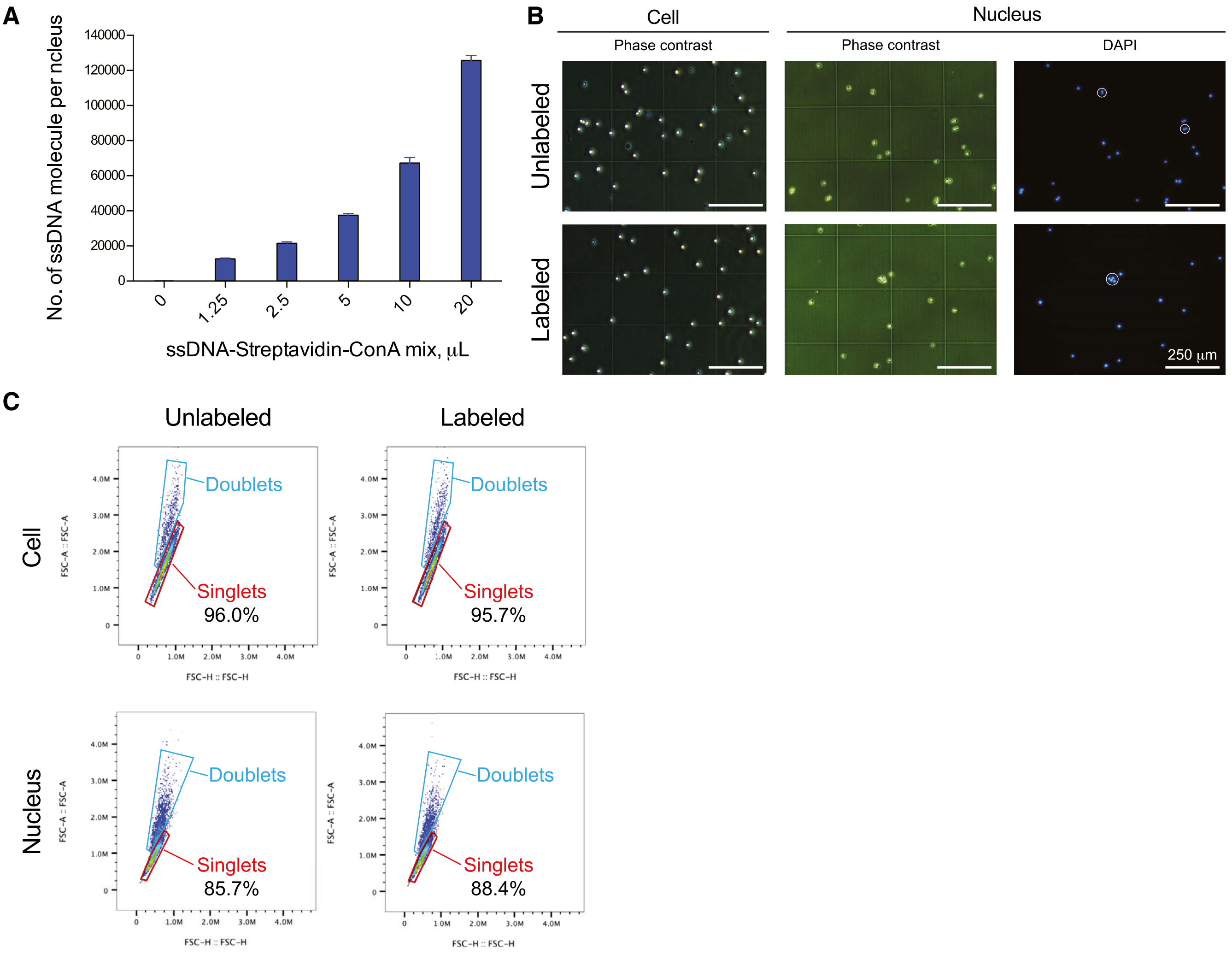

Figure EV1. CASB allows nuclei labeling and does not inducing cell or nucleus aggregation.

A The number of ssDNA molecules immobilized on mESC nuclei was quantified used qPCR. The amount of ssDNA immobilized on nuclei increased with the increased usage of ConA-streptavidin-ssDNA complex and reached at least 120,000 molecules per nucleus. Three independent biological replicates were performed. Error bars represent SD.

B, C Image and flow cytometry analysis of cell and nucleus with or without CASB labeling. Both results demonstrated that ConA-streptavidin-ssDNA complex did not induce cell or nucleus aggregation. White circles highlight nuclear aggregates.

Figure EV2. CASB facilitates scRNA-seq sample multiplexing.

A The poly-A ssDNA molecules immobilized on MEF was detected using RT–qPCR. Both poly-A ssDNA and ActB transcripts can be efficiently capture by RT primer. As expected, barcoding ssDNA can be detected by qPCR even without RT reaction. Three independent biological replicates were performed. Error bars represent SD.

B Heatmap showing the detected relative levels of each CASB barcode in individual cells in scRNA-seq. A total of 12068 cells with sufficient reads were captured; 3,962 cells that contained at least two major barcodes were assigned as cell doublets; 483 cells were assigned as “unlabeled”, as expected due to the inclusion of unlabeled MDA-MB-231 cells.

C Boxplot demonstrating the number of UMI derived from both CASB barcode and mRNA transcripts in cell doublets (n = 3,962) and singlets (n = 7,623). Comparing with singlets, more UMI derived from both CASB barcode and mRNA transcripts were detected in doublets. The central band represents the median; the lower and upper hinges of the boxplot correspond to the first and third quartiles, respectively.

D Scatterplot illustrating a positive correlation between the number of detected UMI from CASB and endogenous transcripts among individual cells. “R” means Pearson’s correlation coefficient. R function “cor.test” was used to obtain the *P*-value.

E Distribution of normalized CABS UMI counts of singlets and individual cell samples. The CASB barcoding manifested a good uniformity of labeling efficiency (5–95 percentile: 2.1–21.8%) (upper panel); comparing with human cell samples, MEF cells had slightly lower labeling efficiency (lower panel).

F Transcriptome-based UMAP comparing labeled and unlabeled untreated MDA-MB-231 cells, in which two cell populations were intermingled.

G Scatterplot demonstrating the well-correlated gene expression profiles between labeled and unlabeled untreated MDA-MB-231 cells. “R” means Pearson’s correlation coefficient.

H t-SNE projection based on the CASB barcode reads captured in scRNA-seq. Cells were colored according to the CASB barcodes, and doublets were marked in black. All 20 sample barcodes can be detected.

I Transcriptome-based UMAP of all MDA-MB-231 cells captured in scRNA-seq. Untreated and 24-h treated cells were highlighted. Cells associated with 24 h-treatment of Niraparib, Rucaparib, and OSI-027 could be well distinguished from untreated cells, whereas those with LCL161 and Fludarabine could not.

© 2021 The Authors

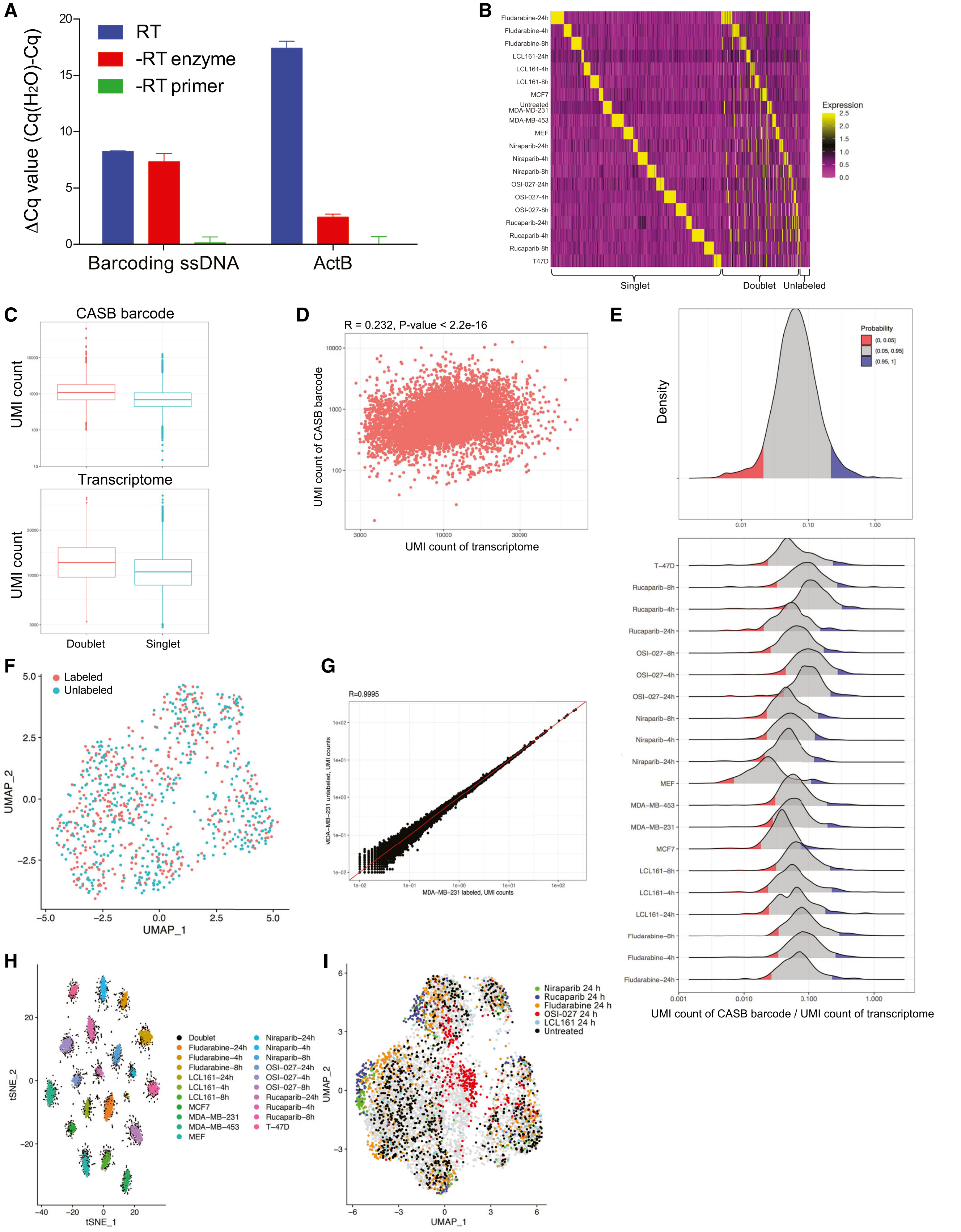
A
RT
-RT enzyme
-RT primer
ΔCq value (Cq(H2O)-Cq)
Barcoding ssDNA
ActB
B
Expression
Singlet
Doublet
Unlabeled
C
CASB barcode
Transcriptome
UMI count
Doublet
Singlet
D
R = 0.232, P-value < 2.2e-16
UMI count of CASB barcode
UMI count of transcriptome
E
Density
Probability
UMI count of CASB barcode / UMI count of transcriptome
F
Labeled
Unlabeled
UMAP_1
UMAP_2
G
R=0.9995
MDA-MB-231 labeled, UMI counts
H
tSNE_1
tSNE_2
I
Niraparib 24 h
Rucaparib 24 h
Fludarabine 24 h
OSI-027 24 h
LCL161 24 h
Untreated
UMAP_1
UMAP_2

Figure EV2.

Figure EV3. Intrinsic heterogeneity of MDA-MB-231 cells.

A, B Transcriptome-based UMAP of untreated and OSI-027-treated MDA-MB-231 cells. Cells were unsupervised clustered and colored into three distinct groups according to the transcriptomic feature revealed by Louvain algorithm (A). UMI count of individual cells was indicated, and no significant difference in UMI distribution was observed between the three clusters as well as between treated and untreated samples (B).

C Neighbor proportion analysis of untreated and OSI-027-treated MDA-MB-231 cells. In cluster 0, untreated cells were distant from treated cells, while, in cluster 1 and 2, untreated cells were 50% neighbored with treated cells.

D Transcriptome-based UMAP of untreated MDA-MB-231 cells. Cells were unsupervised clustered into three distinct groups with Louvain method.

E Transcriptome-based UMAP of untreated and Niraparib- and Rucaparib-treated MDA-MB-231 cells. Cells were unsupervised clustered into three distinct groups with Louvain method.

F Function enrichment analysis of genes that were regulated by OSI-027, Niraparib, and Rucaparib. All three compounds induced expression changes of genes that are enriched in cell death and survival pathway. *P*-values were calculated using Fisher's exact test.

G, H Function enrichment analysis of genes that were commonly up- or downregulated in insensitive cell populations. (G) These genes were highly enriched in the cellular compromise and movement pathways. *P*-value was calculated using Fisher's exact test. (H) Genes that were upregulated in insensitive cell populations and predicted to promote cell movement, including VIM, SQSTM1, NPM1, and RACK1.

© 2021 The Authors

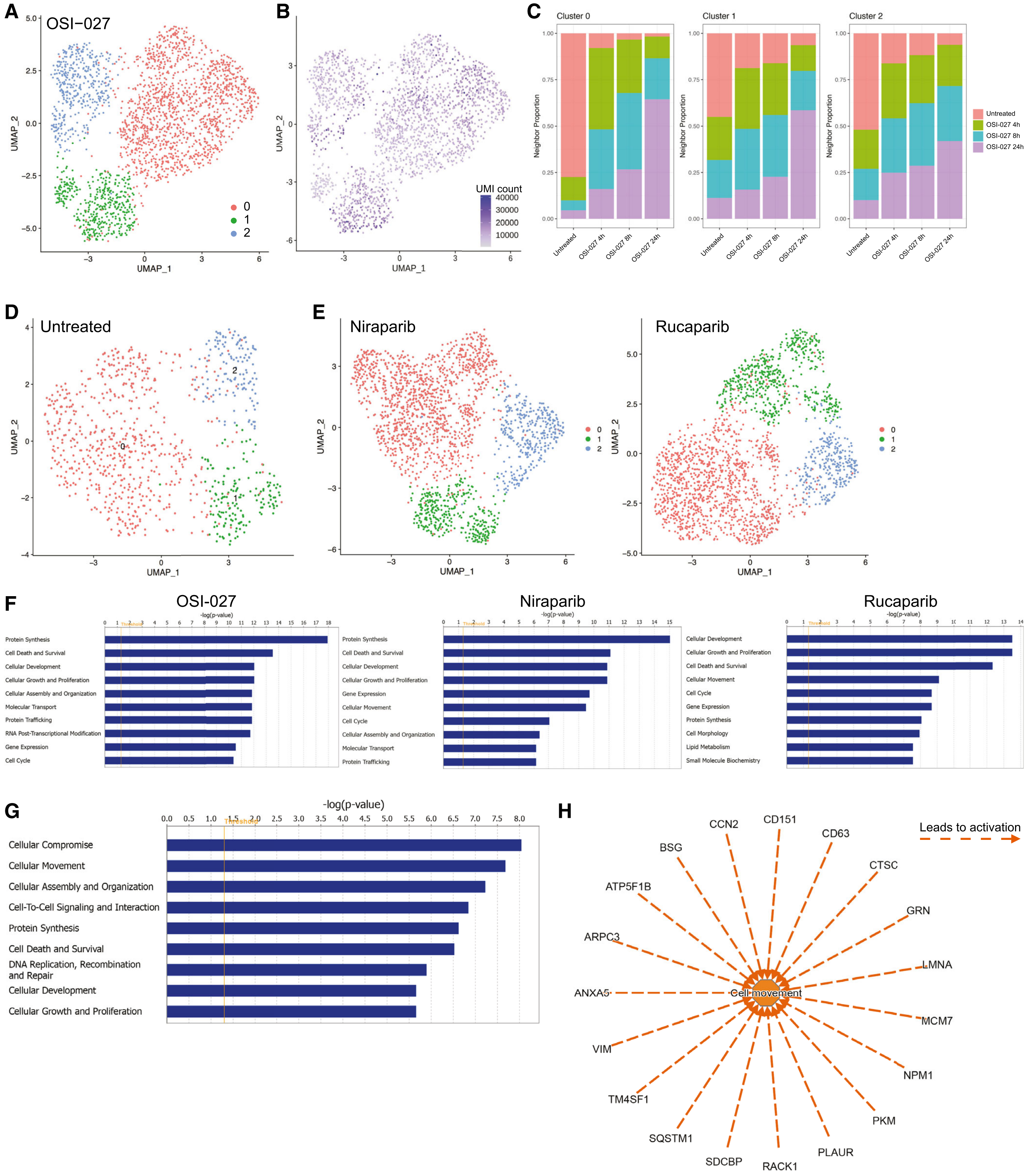
A
OSI−027
B
UMI count
C
Cluster 0
Cluster 1
Cluster 2
Untreated
OSI-027 4h
OSI-027 8h
OSI-027 24h
Neighbor Proportion
D
Untreated
E
Niraparib
Rucaparib
UMAP_1
UMAP_2
F
OSI-027
Niraparib
Rucaparib
-log(p-value)
Protein Synthesis
Cell Death and Survival
Cellular Development
Cellular Growth and Proliferation
Cellular Assembly and Organization
Molecular Transport
Protein Trafficking
RNA Post-Transcriptional Modification
Gene Expression
Cell Cycle
Cellular Movement
Lipid Metabolism
Cell Morphology
Small Molecule Biochemistry
G
-log(p-value)
Threshold
Cellular Compromise
Cellular Movement
Cellular Assembly and Organization
Cell-To-Cell Signaling and Interaction
Protein Synthesis
Cell Death and Survival
DNA Replication, Recombination and Repair
Cellular Development
Cellular Growth and Proliferation
H
Leads to activation
Cell movement
CCN2
CD151
CD63
BSG
CTSC
ATP5F1B
GRN
ARPC3
LMNA
ANXA5
MCM7
VIM
NPM1
TM4SF1
PKM
SQSTM1
SDCBP
RACK1
PLAUR

Figure EV3.

Figure EV4. The three clusters of MDA-MB-231 cells exhibits distinct gene expression pattern.

Heatmap presenting the relative expression levels of cluster-specific genes.

 © 2021 The Authors

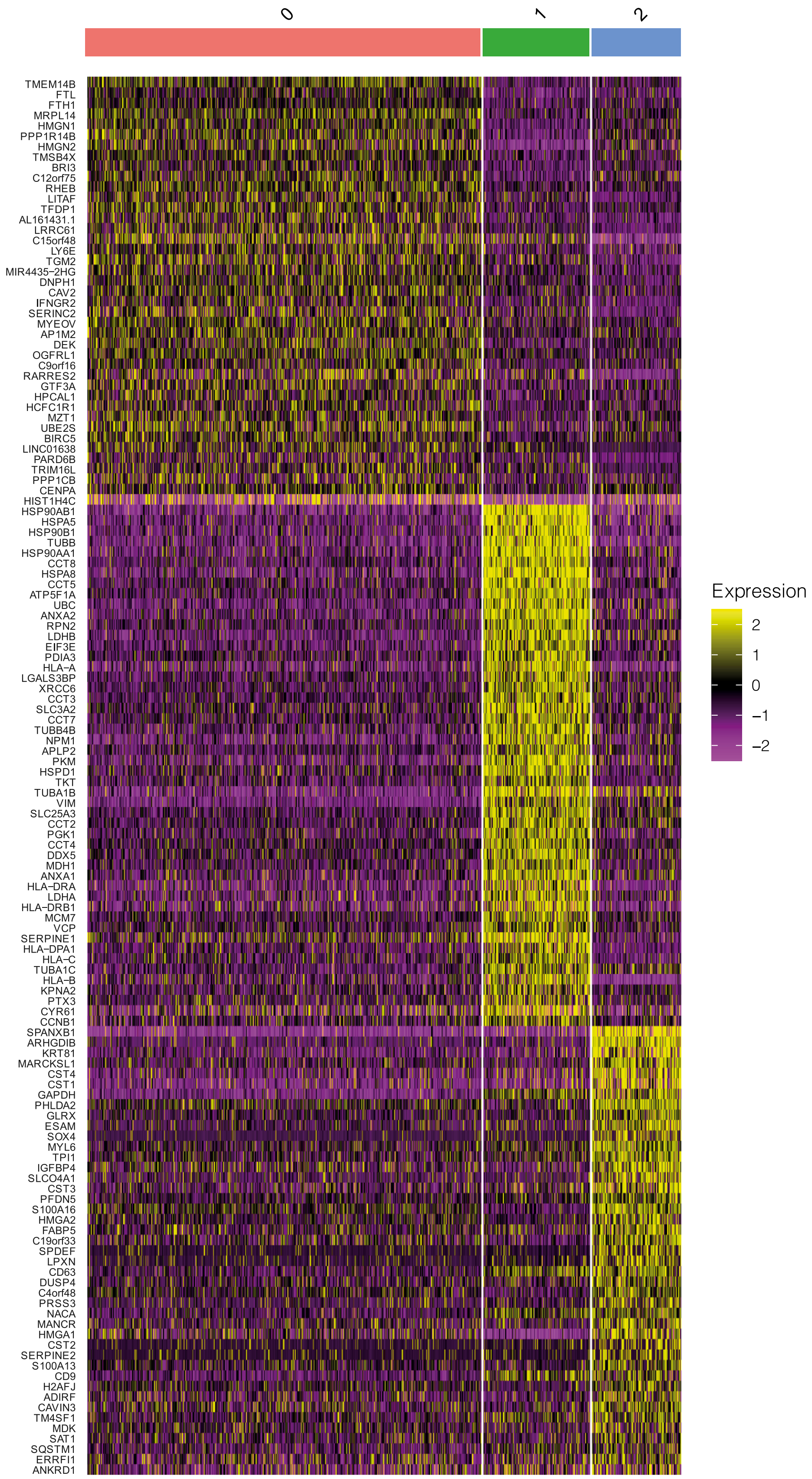
0
1
2
TMEM14B
FTL
FTH1
MRPL14
HMGN1
PPP1R14B
HMGN2
TMSB4X
BRI3
C12orf75
RHEB
LITAF
TFDP1
AL161431.1
LRRC61
C15orf48
LY6E
TGM2
MIR4435−2HG
DNPH1
CAV2
IFNGR2
SERINC2
MYEOV
AP1M2
DEK
OGFRL1
C9orf16
RARRES2
GTF3A
HPCAL1
HCFC1R1
MZT1
UBE2S
BIRC5
LINC01638
PARD6B
TRIM16L
PPP1CB
CENPA
HIST1H4C
HSP90AB1
HSPA5
HSP90B1
TUBB
HSP90AA1
CCT8
HSPA8
CCT5
ATP5F1A
UBC
ANXA2
RPN2
LDHB
EIF3E
PDIA3
HLA−A
LGALS3BP
XRCC6
CCT3
SLC3A2
CCT7
TUBB4B
NPM1
APLP2
PKM
HSPD1
TKT
TUBA1B
VIM
SLC25A3
CCT2
PGK1
CCT4
DDX5
MDH1
ANXA1
HLA−DRA
LDHA
HLA−DRB1
MCM7
VCP
SERPINE1
HLA−DPA1
HLA−C
TUBA1C
HLA−B
KPNA2
PTX3
CYR61
CCNB1
SPANXB1
ARHGDIB
KRT81
MARCKSL1
CST4
CST1
GAPDH
PHLDA2
GLRX
ESAM
SOX4
MYL6
TPI1
IGFBP4
SLCO4A1
CST3
PFDN5
S100A16
HMGA2
FABP5
C19orf33
SPDEF
LPXN
CD63
DUSP4
C4orf48
PRSS3
NACA
MANCR
HMGA1
CST2
SERPINE2
S100A13
CD9
H2AFJ
ADIRF
CAVIN3
TM4SF1
MDK
SAT1
SQSTM1
ERRFI1
ANKRD1
Expression
2
1
0
−1
−2

Figure EV4.

Figure EV5. CASB facilitates snATAC-seq sample multiplexing.

A The number of ATAC-barcode molecules immobilized on mESC nuclei was quantified used qPCR. The amount of ssDNA immobilized on nuclei increased with the increased usage of ConA-streptavidin-ssDNA complex and could reach at least 80,000 molecules per nucleus. Three independent biological replicates were performed. Error bars represent SD.

B Histogram showing the number of CASB barcode reads assigned to individual cells in plate-based snATAC-seq. The cutoff was set as 20,000.

C ATAC-based UMAP comparing labeled and unlabeled HAP1 cells, in which two cell populations were intermingled.

D Scatterplot demonstrating the correlated epigenomic profiles between labeled and unlabeled as well as those between cells collected in plate 1 and 2. The correlation between the labeled and unlabeled cells was similar as that between the two plates. “R” means Pearson’s correlation coefficient. Each dot presents a ATAC peak.

E Heatmap showing the detected relative levels of each CASB ATAC-barcode in individual cells in snATAC-seq. A total of 3,218 cells were obtained with sufficient reads, 305 of which were identified as cell doublets and 23 cells were unlabeled.

F Number of ATAC peaks detected in individual cells from different groups. Each dot represents a cell.

G ATAC-based UMAP of MDA-MB-231 and HAP1 cells. Cells were colored according to the cell line specific barcodes. MDA-MB-231 cells with its specific CASB barcode presented as an isolated cluster.

 © 2021 The Authors

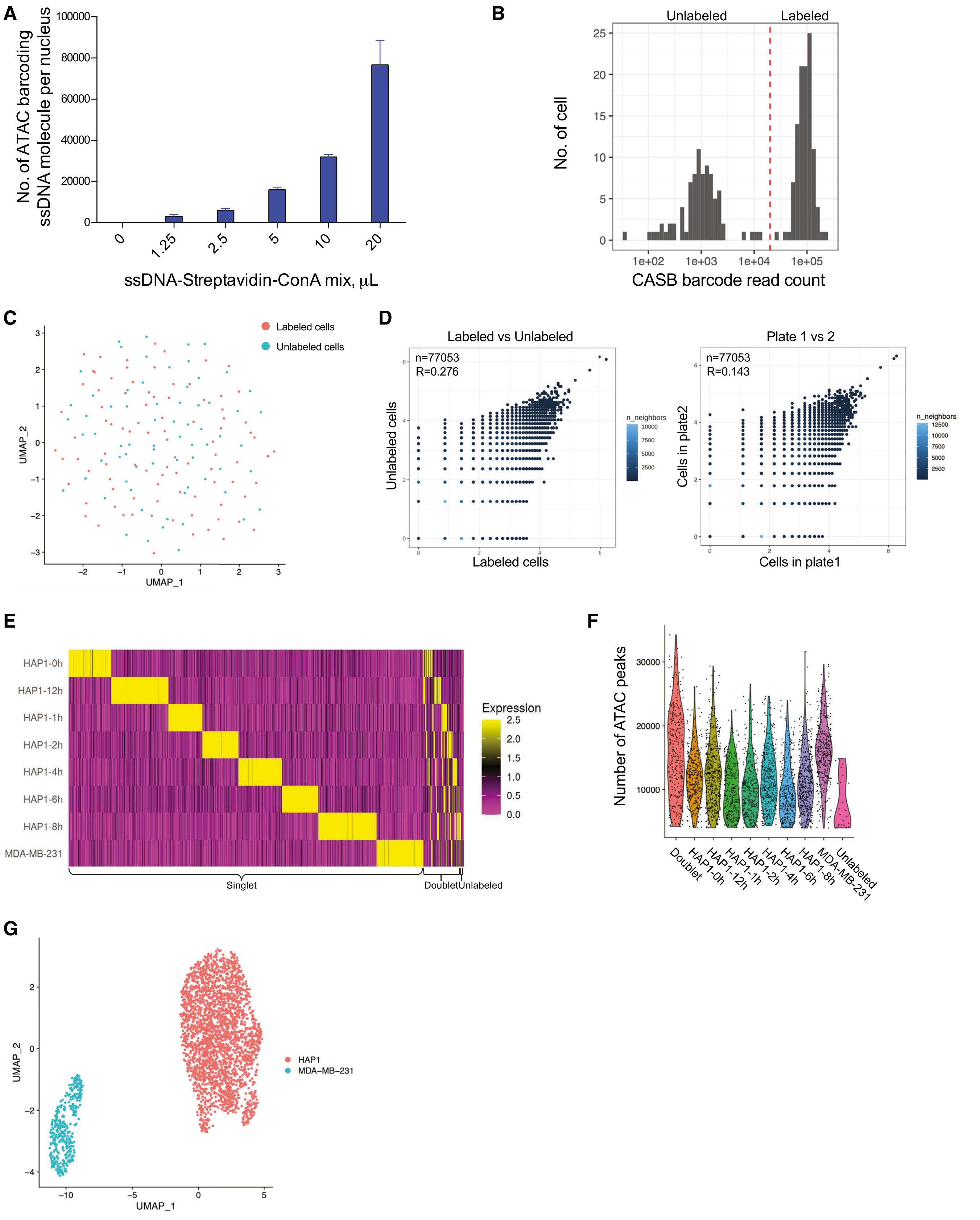

A
No. of ATAC barcoding ssDNA molecule per nucleus
100000
80000
60000
40000
20000
0
0
1.25
2.5
5
10
20
ssDNA-Streptavidin-ConA mix, μL
B
Unlabeled
Labeled
No. of cell
25
20
15
10
5
0
1e+02
1e+03
1e+04
1e+05
CASB barcode read count
C
Labeled cells
Unlabeled cells
UMAP_2
UMAP_1
D
Labeled vs Unlabeled
n=77053
R=0.276
Unlabeled cells
Labeled cells
n_neighbors
Plate 1 vs 2
n=77053
R=0.143
Cells in plate2
Cells in plate1
E
HAP1-0h
HAP1-12h
HAP1-1h
HAP1-2h
HAP1-4h
HAP1-6h
HAP1-8h
MDA-MB-231
Expression
2.5
2.0
1.5
1.0
0.5
0.0
Singlet
Doublet
Unlabeled
F
Number of ATAC peaks
30000
20000
10000
Doublet
HAP1-0h
HAP1-12h
HAP1-1h
HAP1-2h
HAP1-4h
HAP1-6h
HAP1-8h
MDA-MB-231
Unlabeled
G
UMAP_2
UMAP_1
HAP1
MDA−MB−231

Figure EV5.

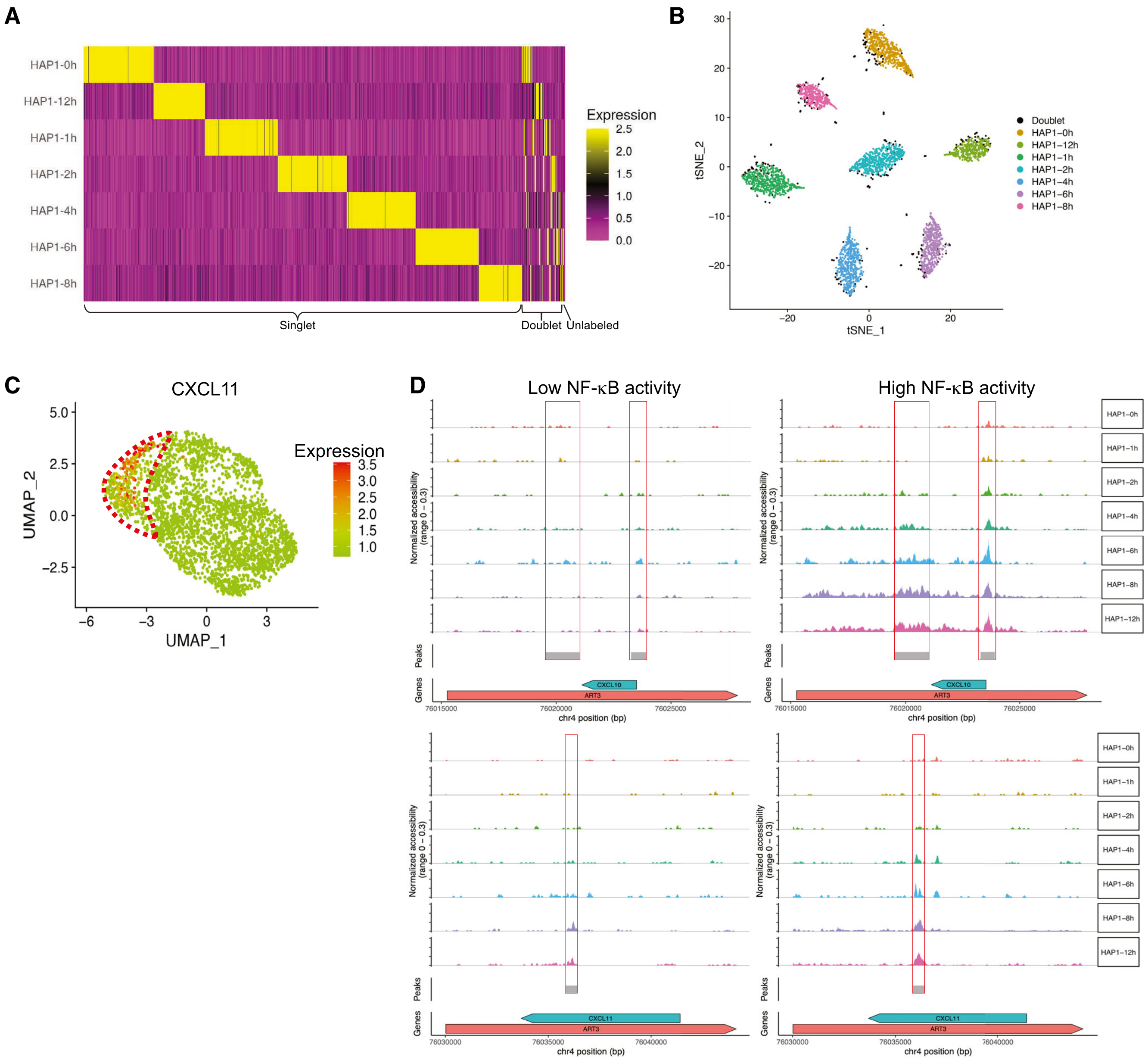

Figure EV6. CASB helps to reveal the dynamic transcriptome change in HAP1 cells.

A Heatmap showing the detected relative levels of each CASB barcode in individual cells in scRNA-seq. A total of 3,407 cells were captured, 294 of which were identified as cell doublets and 9 cells were unlabeled.

B t-SNE projection based on the CASB barcode reads captured in scRNA-seq. Cells were colored according to the CASB barcodes, and doublets were marked in black.

C Transcriptome-based UMAP of HAP1 cells, in which the relative expression of CXCL11 was presented with color code. At later time points, CXCL11 were only actively induced in cluster 2 (circled in red).

D Gene tracks demonstrate the cumulative ATAC signal around CXCL10 and 11 genes in two cell clusters with different NF-kB activity at different time points. Detected ATAC peaks were highlighted by red boxes. Comparing with cells with high NF-kB activity, cells with low NF-kB activity showed limited changes on chromatin accessibility upon INF-γ stimulation.

 © 2021 The Authors

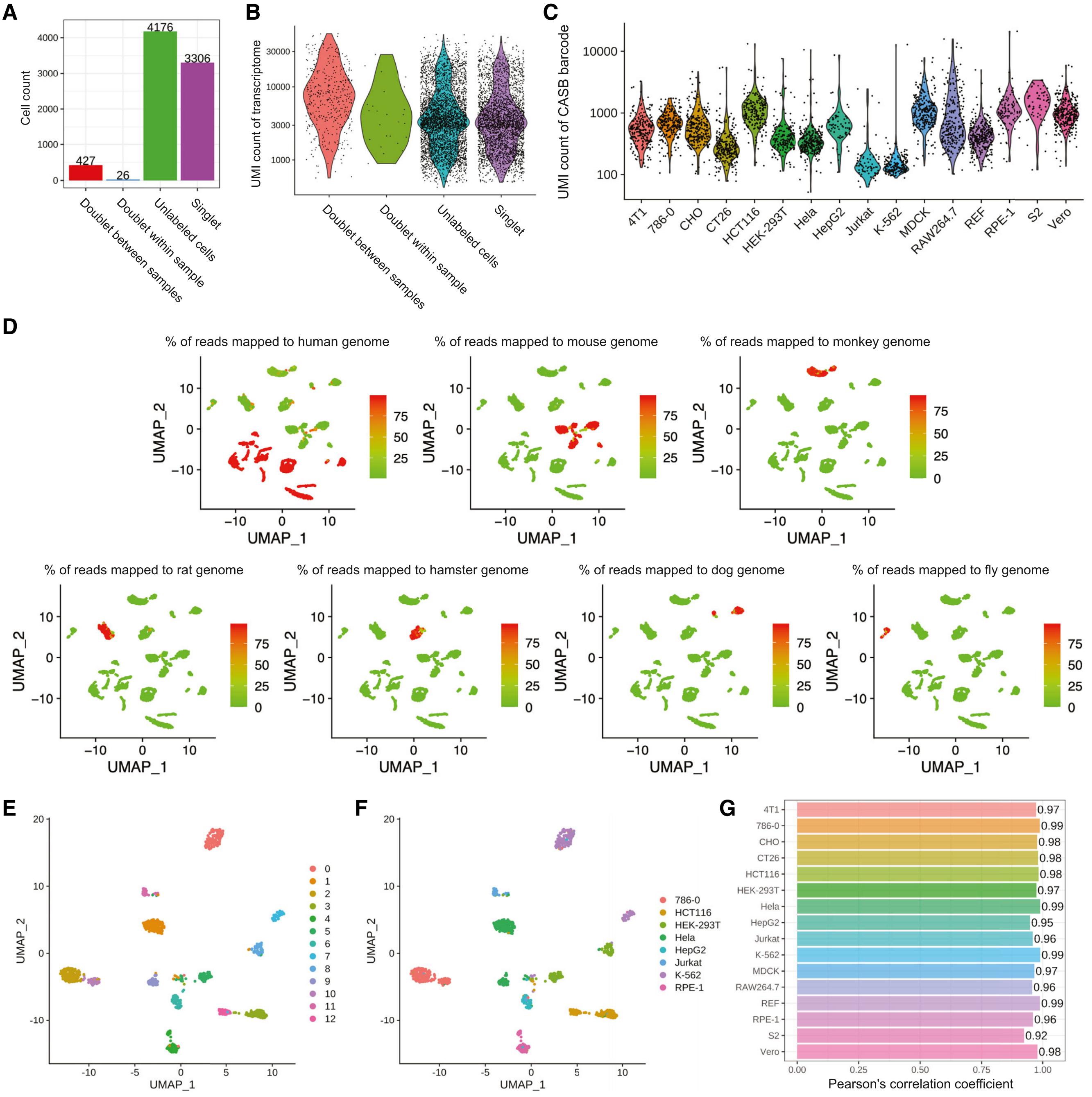

Figure EV7. CASB enables combinatorial indexing.

A Number of cells assigned into different groups.

B Transcriptome UMI count of cells in different groups. Cells assigned as "Doublet" showed relatively higher UMI count. Each dot represents a cell.

C UMI count of CASB barcodes in different cell types. Each dot represents a cell. All 16 cell lines were labeled with sufficient amount of total CASB barcode. The median UMI count of CASB barcode across 16 cell lines ranged from 133 (K-562) to 1,545 (S2), while the variation within individual cell lines ranged from 2.4-fold (K-562) to 19.4-fold (RAW264.7) (5–95 percentile).

D Transcriptome-based UMAP of all cell singlets, in which cells were unsupervised clustered. Cells were colored based on the percentage of reads mapped to indicated genome.

E, F Transcriptome-based UMAP of eight human cell lines, in which cells were unsupervised clustered. Cells were colored according to the transcriptomic feature revealed by Louvain algorithm (E) and CASB barcode combinations (F).

G Pearson's correlation coefficient was calculated for cumulative gene expression profiles between labeled and unlabeled cells of different cell types, showing a negligible influence of CASB labeling on transcriptome profile.

© 2021 The Authors
